# Polymeric Nanoparticles for Retinal Drug Delivery

**DOI:** 10.18502/jovr.v19i1.15417

**Published:** 2024-03-14

**Authors:** Mazda Rad-Malekshahi

**Affiliations:** ^1^Department of Pharmaceutical Biomaterials and Medical Biomaterials Research Center, Faculty of Pharmacy, Tehran University of Medical Sciences, Tehran, Iran

The introduction of drugs that inhibit vascular endothelial growth factor (VEGF) has revolutionized treatment for ocular disorders secondary to angiogenesis such as choroidal or corneal neovascularization. Studies have shown that injection of these agents into the vitreous can reduce medication doses by 500-fold as compared to intravenous injection. However, due to the short half-life of these agents in the vitreous, frequent intraocular injections are required to maintain their therapeutic effects. Such repetitive injections may entail mild to serious complications including patient non-compliance, intraocular hemorrhage, cataracts, retinal detachments and endophthalmitis.^[1,2]^

Sustained and controlled drug delivery systems have shown promising performance in addressing these problems. In this regard, a wide range of nanoparticles have been introduced as safe and efficient systems for drug delivery. The use of these carriers serving as nano-reservoirs for VEGF-inhibitors can be an effective strategy to reduce the number of intraocular injections. Moreover, decreased enzymatic degradation, increased drug solubility, and improved drug bioavailability can be achieved by nanoparticulate delivery systems. Due to the capability of retinal pigment epithelium (RPE) cells to phagocytose nanoparticles, these drug carriers can also perform as efficient retinal drug delivery systems for posterior segment diseases. Several nano-structured formulations have been studied during the last decades among which biodegradable polymers have attracted increasing interest due to biocompatibility, non-toxicity, and diversity in physicochemical properties.^[3,4,5]^

In this issue of *Journal of Ophthalmic and Vision Research*, Chaharband and coworkers have investigated the preparation and *in vivo* evaluation of propranolol-loaded nanoparticles for treatment of choroidal neovascularization (CNV).^[6]^ Since the therapeutic potential of propranolol has been demonstrated in previous studies,^[7,8]^ Chaharband et al focused on extending the presence of the drug in the vitreous following intravitreal injections in rabbits. The strength of this study and similar previous studies from this group is their precise evaluation of drug pharmacokinetics and comparison between free and loaded drug concentrations in nanoparticles. ${\;}^{\left[8,10\right]}$ However, the study was limited by certain issues such as the number of animals, difficulties in vitreous sampling and inter-animal differences. Further studies in the future may provide more information in this area of research. A noteworthy finding of this study was demonstration of the capability of polymeric nano-carrier, poly(lactic-co-glycolic) acid, to prolong the presence of propranolol in the vitreous (mean residence time) and reduce the frequency of intraocular injections. The *in vivo* results of their study demonstrated the retinal uptake of nanoparticles and persistence of the drug in the vitreous and posterior segment ocular tissues. In summary, the study by Chaharband and colleagues introduces a new approach for the treatment of CNV which can be considered for future studies in this field.

##  Financial Support and Sponsorship

None.

##  Conflicts of Interest

None.
